# *Vibrio cholerae*: a fundamental model system for bacterial genetics and pathogenesis research

**DOI:** 10.1128/jb.00248-24

**Published:** 2024-10-15

**Authors:** Julia C. van Kessel, Andrew Camilli

**Affiliations:** 1 Department of Biology, Indiana University, Bloomington, Indiana, USA; 2 Tufts University, School of Medicine, Boston, Massachusetts, USA; Geisel School of Medicine at Dartmouth, Hanover, New Hampshire, USA

**Keywords:** *Vibrio*, *Vibrio cholerae*, virulence factors, pathogenesis, genetic competence, quorum sensing, toxins

## Abstract

Species of the *Vibrio* genus occupy diverse aquatic environments ranging from brackish water to warm equatorial seas to salty coastal regions. More than 80 species of *Vibrio* have been identified, many of them as pathogens of marine organisms, including fish, shellfish, and corals, causing disease and wreaking havoc on aquacultures and coral reefs. Moreover, many *Vibrio* species associate with and thrive on chitinous organisms abundant in the ocean. Among the many diverse *Vibrio* species, the most well-known and studied is *Vibrio cholerae*, discovered in the 19th century to cause cholera in humans when ingested. The *V. cholerae* field blossomed in the late 20th century, with studies broadly examining *V. cholerae* evolution as a human pathogen, natural competence, biofilm formation, and virulence mechanisms, including toxin biology and virulence gene regulation. This review discusses some of the historic discoveries of *V. cholerae* biology and ecology as one of the fundamental model systems of bacterial genetics and pathogenesis.

## INTRODUCTION

**Fig 1:**
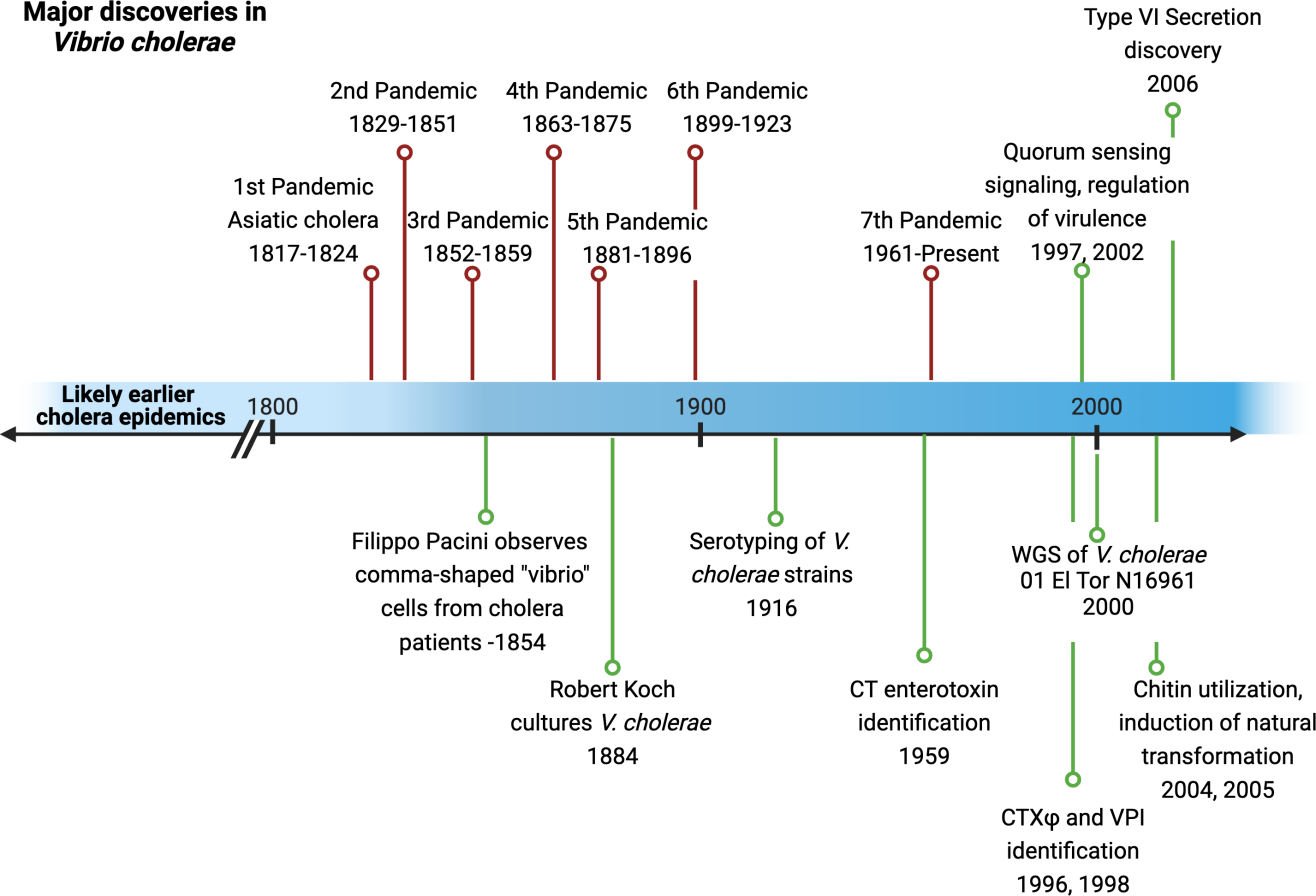
Timeline of major discoveries in *Vibrio cholerae* and cholera.

Those of us who study *Vibrio* species in our laboratories know that most people have only heard of one *Vibrio* – the causative agent of the disease cholera. *V. cholerae* is one of the more infamous human pathogens of modern times, yet it has likely plagued humankind for thousands of years (1, 2) (Fig. 1). Cholera is contracted when people consume water contaminated with *V. cholerae* bacteria. Once these cells survive passage through the stomach’s acidic environment, they colonize the small intestine, delivering a toxin to the host epithelium that drives the copious release of ions and water into the gut lumen. The resulting profuse secretory diarrhea results in severe fluid volume depletion, which may lead to circulatory collapse and death. Standard and effective treatment, if available, is intravenous or oral hydration. Although seemingly a simple treatment, many outbreaks of cholera are linked to poor sanitation conditions or catastrophic events, thus precluding the availability of treatment in many affected regions. The World Health Organization (WHO) describes the current situation worldwide as an “upsurge of the 7th pandemic,” with numerous ongoing outbreaks reported and efforts being coordinated to perform surveillance, vaccination, prevention, case management, and increased assessment and response actions (3).

Although several regions of the world struggle with the ongoing 7th pandemic of cholera that began in 1961, many bacteriologists have latched onto *V. cholerae* as an organism for studying mechanisms of bacterial behaviors, genetics, ecology, evolution, and more. Here, we describe some of the discoveries in the *V. cholerae* field that led to this comma-shaped bacterium becoming a fundamental model organism for bacterial research.

## THE BEGINNING OF THE SCIENTIFIC STUDY OF *V. CHOLERAE*

Likely, cholera originated thousands of years ago, given that the Sanskrit word “visuchika,” which accurately describes cholera symptoms, was used as early as 400 B.C.E. (1, 2). In modern times, the first recorded pandemic began in 1817 and ended around 1824. There have since been six more pandemics, with the ongoing 7th pandemic showing no signs of dwindling. The second half of the 19th century saw momentous discoveries in the cholera field. In 1853, during the 3rd pandemic, Filippo Pacini used his microscopy expertise to view and describe for the first time the presence of comma-shaped bacilli, which he named *Vibrio cholera*, in samples from people who had died of cholera. In 1884, Robert Koch’s bacterial cultivation techniques enabled him to successfully grow *V. cholerae* on solid media (first on sliced potatoes, later on agar), and he detailed its phenotypical characteristics (4). In 1855, John Snow diligently described and documented the 1854 London cholera outbreak and correctly proposed the spread of the disease through contaminated water (5). This discovery was revolutionary because it was dogma at the time that cholera was caused by bad air.

Decades later, in 1916, the initial “fingerprinting” of *V. cholerae* strains began, in which the characteristics of each strain were used to classify subtypes (6). Hemolysis or hemagglutination activity and phage-typing were the earliest technologies to be utilized until the onset of the molecular and genomic era enabled more accurate approaches: PCR, chromosome mapping, and eventually whole genome sequencing (7).

Here, we briefly review some seminal works from modern times on *V. cholerae* to highlight its broad impact as a model organism.

## TOXIN BIOLOGY

Among the many discoveries of novel molecular biology and pathogenesis mechanisms in *V. cholerae*, one of the first and most famous is identifying cholera toxin (CT). Groundbreaking experiments published by Sambhu Nath De and colleagues in the 1950s using the rabbit ligated loop model system revealed that the toxic effects of *V. cholerae* target the intestinal mucosa, causing fluid secretion (8–12). Their seminal paper in 1959, showing that the effector of these phenotypes could be derived from *V. cholerae* culture supernatants, led to many more studies that unveiled the existence of CT (8). Later, CT was purified and characterized *in vitro* and assayed *in vivo*, showing that it comprises two peptide subunits, A (toxic-active) and B (binding), that interact with the GM1 ganglioside receptor on host intestinal cell membranes (13–19). Decades of molecular, genetic, biochemical, structural, immunological, and cell biological studies of CT and its effects on the host provided models for enterotoxins in other organisms, paradigm shifts in vaccine development, and cholera disease treatment (20).

## IDENTIFICATION OF VIRULENCE GENES AND THEIR REGULATION

Notably, the vast majority of the >200 *V*. *cholerae* serogroups do not produce CT and thus do not cause cholera. One serogroup, O1 (divided into O1 El Tor and classical biotypes) is believed to be responsible for all seven recorded pandemics (21). Some, but not all strains of the O1 serogroup, cause disease because they carry specific virulence genes, including the CTXΦ prophage, which encodes the CT-coding genes *ctxA* and *ctxB*. The discoveries of the CTXΦ and *Vibrio* pathogenicity islands (VPIs) encoding other key virulence factors marked another paradigm shift in understanding cholera biology and pathogenesis Box 1 (22–34). Multiple signal transduction circuits were uncovered that enable *V. cholerae* to sense and respond to external stimuli, such as bile, salt, pH, and temperature (35). These pathways drive control of virulence genes via ToxR, which, together with ToxS, directly or indirectly regulate major virulence genes: *ctxAB* and the genes encoding the major colonization factor and receptor for the CTXΦ phage, the toxin co-regulated pilus (TCP), among others (22–24, 26, 32, 33).

Box 1. **Unraveling the mysteries of cholera: the legacy of Ron Taylor**

For three decades (1986–2016), the late Ronald K. Taylor led an incredibly productive genetics-based investigation into the heart of cholera — How *V. cholerae* turns on virulence genes upon entry into the human GI tract and how it colonizes the small intestine. During this period, he trained numerous scientists in the classroom and laboratory, as well as in Cold Spring Harbor’s Advanced Bacterial Genetics course. He also contributed important genetic tools and methodologies to the field. He first learned genetic sleuthing during his graduate training with Thomas Silhavy at the University of Maryland Baltimore. Ron, with his postdoctoral mentor, John Mekalanos, at Harvard Medical School, then turned his attention to unveiling the mysteries of *V. cholerae*. Ron’s discoveries are too many and varied to list all, but some highlights vis-à-vis *V. cholerae* virulence include:

**Table:** 

1987–1989	Toxin-coregulated pilus (TCP) is the major virulence factor (36). Tn*phoA*-mediated discovery of TCP pilin and other exported virulence factors (22, 37). Defined the ToxR binding site within the cholera toxin operon promoter (23, 38).
1990	Anti-TCP antibodies protect against infection (39).
1992	TcpG catalyzes disulfide bond formation in multiple virulence proteins (40).
1995–2000	Identified key residues on TCP for microcolony formation, adherence, and antigenicity (41–43).
1997–2000	cAMP-CRP and H-NS repress virulence genes outside the host (44, 45). Identification of environmental signals controlling virulence gene regulation (35).
2003–2011	TcpF is a TCP-dependent secreted factor that is essential for colonization (46–49). GbpA binds chitin in the environment and receptors in the small intestine (50).

## CHITIN UTILIZATION, NATURAL COMPETENCE, AND ECOLOGY

Chitin is the most abundant polymer in the ocean (51). Many organisms, including *V. cholerae*, have evolved to utilize chitin as a carbon and nitrogen source. *V. cholerae* is typically associated with chitinous surfaces in its aquatic lifestyle and reservoir. The influence of chitin on *V. cholerae* catabolism, ecology, and genetic exchange became apparent in the 2000s when it was discovered that growth on chitin induced the expression of a suite of genes for chitin utilization and natural competence (52). This discovery has been further explored, revealing a variety of molecular mechanisms, including chitin sensing, chitin-binding pilus, chitin uptake, regulation of downstream genes, DNA-binding pilus, DNA uptake, and recombination, and the impact of chitin on other systems, such as type VI secretion (53). The chitin-regulated processes extend beyond catabolism to genetic transfer and evolution, chemotaxis, biofilm formation, community structure, symbiotic and pathogenic relationships, nutrient cycling, and more (51). Studies of *V. cholerae* and the role of chitin on its biophysiology are broadly important to our understanding of the complex evolutionary history of water-borne, facultative pathogens.

## QUORUM SENSING

Much of the early work uncovering the bacterial cell–cell communication process called quorum sensing was performed in two *Vibrio* species: *Vibrio fischeri* and *Vibrio harveyi* (54–67). Of note, the strain *V. harveyi* BB120 (with which most studies referred to herein were performed) was later re-classified as *Vibrio campbellii* (68). This work was sparked by the ability of these two organisms to produce bioluminescence regulated by quorum sensing, thus providing an easily accessible phenotype to study the genetics and chemistry of the quorum-sensing signaling pathways. As *Vibrio* autoinducer signals and receptors were discovered in *V. campbellii* and *V. fischeri*, new experiments showed that other *Vibrio* species, including *V. cholerae*, could signal to *V. campbellii* (69). Soon afterward, homologs of quorum-sensing system components as key virulence regulators were discovered in *V. cholerae* (70). Indeed, subsequent studies revealed that quorum sensing regulates the genes that control biofilm formation and virulence, including biofilm activators VpsR and VpsT, protease HapA, transcription activators AphA/AphB, and virulence activators TcpPH and thus ToxT (reviewed further in (70–77). Identifying the *V. cholerae*-specific autoinducer CAI-1 structure paved the way for many studies of quorum-sensing signals, receptor specificity, and virulence gene regulation in *V. cholerae* (78).

## CONTACT-DEPENDENT KILLING

The discovery of type VI secretion systems (T6SSs) in *V. cholerae* marks another paradigm shift in our understanding of bacterial interactions. In 2006, it was revealed that *V. cholerae* could fend off predation by the protist *Dictyostelium,* and the T6SS encoding *vas* genes responsible were identified (79). Using a combination of approaches, the field determined the structure of T6SS “nanomachine,” observed its live assembly, firing, and disassembly, and the effects of delivery of effector proteins into target cells (80–85). Later studies revealed the regulatory mechanisms controlling T6SS, the broad conservation of T6SS among Gram-negative bacteria, and the impact of T6SS on horizontal gene transfer, leading to evolution and divergence in T6SS repertoires (86–92). The now-clear role of T6SS in competition between bacterial species within mixed communities was found to drive competition in numerous niches beyond those occupied by *V. cholerae*. As examples, these include (1) symbiotic interactions, such as *V. fischeri* interstrain competitions that drive strain colonization in the squid *Euprymna scolopes* (93), (2) pathogenic interactions, such as the competition between *Agrobacterium tumefaciens* with *Pseudomonas aeruginosa* in plant infections (94), and (3) microbe–microbe interactions in environmental niches, such as the competitions between *Bacteriodales* in mammalian gut microbiota (95).

## FUTURE OUTLOOK

The research on *V. cholerae* establishes this microbe as an exemplary model organism that continues to foster broad research across disciplines. The reasons that *V. cholerae* became a model microbe include its short generation time, simple requirements for growth, efficient genetic tools, and a long history of host biology coupled with clinical studies. *V. cholerae* research continues to expand and has become a model for additional fields, such as chromosome replication and organization, host signaling impacts on pathogenesis, phage/host arms race, and more. Although many microbes have served microbiology in notable capacities, it is arguable that *V. cholerae* has earned its place among the “greats.”
